# Computational Drug Repositioning for Chagas Disease Using Protein-Ligand Interaction Profiling

**DOI:** 10.3390/ijms21124270

**Published:** 2020-06-16

**Authors:** Alfredo Juárez-Saldivar, Michael Schroeder, Sebastian Salentin, V. Joachim Haupt, Emma Saavedra, Citlali Vázquez, Francisco Reyes-Espinosa, Verónica Herrera-Mayorga, Juan Carlos Villalobos-Rocha, Carlos A. García-Pérez, Nuria E. Campillo, Gildardo Rivera

**Affiliations:** 1 Laboratorio de Biotecnología Farmacéutica, Centro de Biotecnología Genómica, Instituto Politécnico Nacional, Reynosa 88710, Mexico; ajuarezs1500@gmail.com (A.J.-S.); frelibi@hotmail.com (F.R.-E.); veronica_qfb@hotmail.com (V.H.-M.); jc.villalobosrocha@yahoo.com (J.C.V.-R.); 2 Biotechnology Center (BIOTEC), Technische Universität Dresden, 01307 Dresden, Germany; michael.schroeder@tu-dresden.de (M.S.); sebastian.salentin@biotec.tu-dresden.de (S.S.); joachim.haupt@biotec.tu-dresden.de (V.J.H.); 3 Departamento de Bioquímica, Instituto Nacional de Cardiología Ignacio Chávez, Ciudad de Mexico 14080, Mexico; emma_saavedra2002@yahoo.com (E.S.); cita2812@gmail.com (C.V.); 4 Departamento de Ingeniería Bioquímica, Unidad Académica Multidisciplinaria Mante, Universidad Autónoma de Tamaulipas, Mante 89840, Mexico; 5 Scientific Computing Research Unit, Helmholtz Zentrum München, 85764 Neuherberg, Germany; carlosagp@hotmail.com; 6 Centro de Investigaciones Biológicas (CIB-CSIC), Ramiro de Maeztu 9, 28040 Madrid, Spain; nuria.campillo@csic.es

**Keywords:** antiprotozoal, chagas disease, FDA-Drugs, molecular docking, protein-ligand interaction profiler, repositioning

## Abstract

Chagas disease, caused by *Trypanosoma cruzi* (*T. cruzi*), affects nearly eight million people worldwide. There are currently only limited treatment options, which cause several side effects and have drug resistance. Thus, there is a great need for a novel, improved Chagas treatment. Bifunctional enzyme dihydrofolate reductase-thymidylate synthase (DHFR-TS) has emerged as a promising pharmacological target. Moreover, some human dihydrofolate reductase (*Hs*DHFR) inhibitors such as trimetrexate also inhibit *T. cruzi* DHFR-TS (*Tc*DHFR-TS). These compounds serve as a starting point and a reference in a screening campaign to search for new *Tc*DHFR-TS inhibitors. In this paper, a novel virtual screening approach was developed that combines classical docking with protein-ligand interaction profiling to identify drug repositioning opportunities against *T. cruzi* infection. In this approach, some food and drug administration (FDA)-approved drugs that were predicted to bind with high affinity to *Tc*DHFR-TS and whose predicted molecular interactions are conserved among known inhibitors were selected. Overall, ten putative *Tc*DHFR-TS inhibitors were identified. These exhibited a similar interaction profile and a higher computed binding affinity, compared to trimetrexate. Nilotinib, glipizide, glyburide and gliquidone were tested on *T. cruzi* epimastigotes and showed growth inhibitory activity in the micromolar range. Therefore, these compounds could lead to the development of new treatment options for Chagas disease.

## 1. Introduction

American trypanosomiasis, also known as Chagas disease, is a protozoan infectious disease caused by the parasite *Trypanosoma cruzi (T. cruzi*). It affects approximately eight million people, mainly in Latin America. *T*. *cruzi* is transmitted in the feces of a triatomine vector, during blood meal intake. Other mechanisms of transmission include blood transfusion, organ transplantation and congenital transmission [1].

Chagas disease is usually asymptomatic in the acute phase; however, some common symptoms such as fever and portal-of-entry effects can occur. In the chronic phase, cardiac disease affects around 30−40% of patients who suffer arrhythmias, tachycardia, heart failure and sudden death [2].

Current pharmacological treatment for Chagas disease is based on nifurtimox and benznidazole. These drugs are effective in the acute phase, but show limited activity in the chronic phase [3]. Additionally, these drugs require constant monitoring to reduce risks derived from their side effects, mainly skin disorders and abdominal pain [4]. Due to the difficulties related to toxicity and drug resistance, new anti-Chagas drugs are needed [5].

In the last two decades, drug repositioning has been an excellent strategy to develop new drugs in a shorter time and with lower costs [6]. Examples of drug repositioning for Chagas treatment are posaconazole and ravuconazole, which entered phase II clinical trials. Unfortunately, they showed poor results compared to benznidazole. However, combination therapy could lead to better results [7]. Drug repositioning combines available knowledge from different sources to repurpose drugs for new indications [6]. Multiple approaches related to drug repositioning have been used to identify new anti-Chagas disease agents. Generally, they can be divided into in vitro/in vivo screening [8,9,10,11], literature-based [12,13], and computational studies [14,15,16]. In this latter field, molecular docking has proved to be a powerful tool. It has been used in several inhibitor discovery studies [17,18,19,20] to understand the potential mechanisms of inhibition and to display the nature of the molecular interactions between an active compound and its target. In this context, structural information is crucial, and protein crystallographic data is the basis for structure-based studies.

Several *T. cruzi* enzymes have been identified as therapeutic targets [21,22,23,24,25,26]. Currently, there are twelve validated *T*. *cruzi* drug targets with at least one crystal reported in the Protein Data Bank (PDB) [14]. In this work, we propose the repositioning of FDA-approved drugs for the inhibition of the bifunctional enzyme dihydrofolate reductase-thymidylate synthase (DHFR-TS) of *T. cruzi*. This bifunctional enzyme catalyzes the reduction of folate to tetrahydrofolate and the subsequent synthesis of thymidylate, an essential precursor in the synthesis of DNA [27]. For this reason, dihydrofolate reductase (DHFR) and thymidylate synthase (TS) have been used as targets in the treatment of several diseases such as cancer [28,29] and infections [30,31,32]. In the case of *T. cruzi* DHFR-TS (*Tc*DHFR-TS), several antifolates have shown inhibitory activity: trimetrexate, methotrexate and pyrimethamine [32]. In order to identify new anti-Chagasic compounds, a food and drug administration (FDA)-approved drug library was virtually screened against *Tc*DHFR-TS. This screening consisted of a novel approach combining docking studies and an interaction profile comparison.

## 2. Results

### 2.1. Assessment of Trimetrexate-TcDFHR-TS Complex

**Figure 1:**
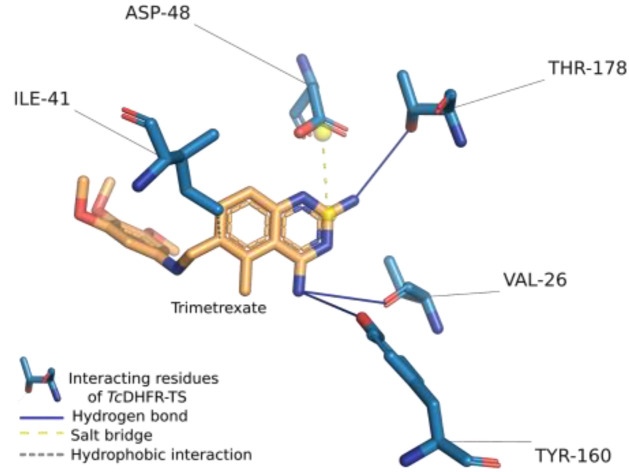
Trimetrexate interactions in complex with *Trypanosoma cruzi* dihydrofolate reductase-thymidylate synthase (*Tc*DHFR-TS). The crystal structure of the complex was analyzed using the protein-ligand interaction profiler (PLIP).

To determine the non-covalent interactions and other interactions of the trimetrexate-*Tc*DHFR-TS complex, the protein-ligand interaction profiler (PLIP) was used to analyze the crystal structure of the available structural complex (PDB ID: 3HBB). PLIP is a widely used tool that determines the molecular interactions between a ligand and its target [33]. Figure 1 shows the interactions calculated by PLIP of the A chain of 3HBB, as well as the conformation of the ligand and the interacting protein residues in the complex. It can be seen that the orientation of trimetrexate in this complex suggests that only one side of the structure influences the inhibition mechanism of *Tc*DHFR-TS. Trimetrexate features a quinazoline with two amine groups and a trimethoxyaniline moiety. The quinazoline group is oriented towards interacting residues of *Tc*DHFR-TS; their amines interact with THR-178 and VAL-26 through hydrogen bonds. This group also forms a salt bridge between its pyrimidine-ring and ASP-48. The authors of this crystal structure point out that the active-site is mainly hydrophobic [34]. In this study, PLIP could only determine a hydrophobic interaction between trimetrexate and ILE-41. Nevertheless, PLIP is a deterministic method that reduces the number of interaction contacts to avoid the bias for strong influences of hydrophobic interactions.

PLIP analysis also provided the ligand properties and the properties of the binding site residues the ligand was near to or interacted with. These properties and the noncovalent interactions of the complex were crucial information for understanding the molecular nature of *Tc*DHFR-TS inhibition. The features calculated were classified into three categories and are summarized in Table 1. The first category, was ligand properties, which were the number of hydrogen bond donors and acceptors, rings, and the number of rotatable bonds in the structure. The next category was based on binding site residues, where properties such as charge, polarity, aromaticity, size, and others were included. Finally, interactions were considered in two ways, type of interaction and type of interaction with a specific residue.

### 2.2. Redocking

**Figure 2:**
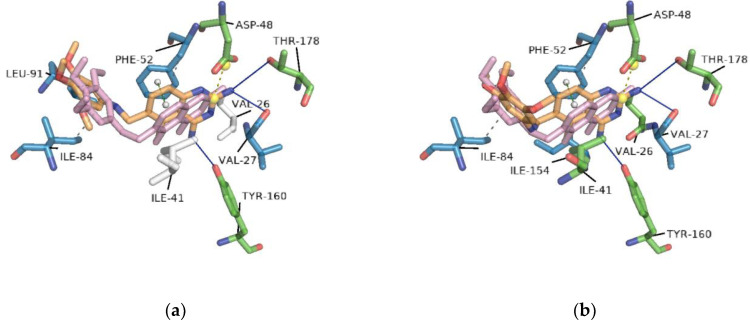
Docking conformations of trimetrexate in complex with *Tc*DHFR-TS: (**a**) conformation with the lowest free energy of binding; (**b**) conformation with the lowest root-mean-square deviation (RMSD). Docking was performed on the active site using vina. Residues in blue interacted with the docking conformation. In green, residues that interacted in both docking and crystal conformation. White residues are those that only interacted with the crystal conformation.

For molecular docking analysis, the AutoDock Vina (vina) program was used. To assess the docking protocol and reproducibility of the binding conformation observed in the crystal structure, trimetrexate and NADPH were docked separately against *Tc*DHFR-TS; this strategy is called redocking. In this redocking, nine different conformations of trimetrexate were generated, a default parameter of vina. Figure 2 presents two of these conformations and highlights the differences between crystallized and docking generated conformations based on the interactions calculated with PLIP. The conformation with the lowest free energy of binding shared interactions with only three of the five interacting residues showed in the crystal analysis mentioned before. On the other hand, the conformation with the lowest root-mean-square deviation (RMSD) showed interactions with the same amino acids as the crystal conformation. Table 2 summarizes the docking conformations of trimetrexate.

In the case of NADPH, cofactor of *Tc*DHFR-TS, the conformation with the highest vina score (−10.0 Kcal/mol) had the lowest RMSD, 0.846 Å. This ligand interacted by hydrogen bonding with ALA-28, ILE-35, ASP-37, GLY-38, ARG-39, ARG-78, LYS-79, THR-80, SER-101, GLY-157, SER-158 and VAL-159. It had hydrophobic contacts with ILE-41, THR-80 and TYR-160.

### 2.3. Interaction Profile Generation

**Figure 3:**
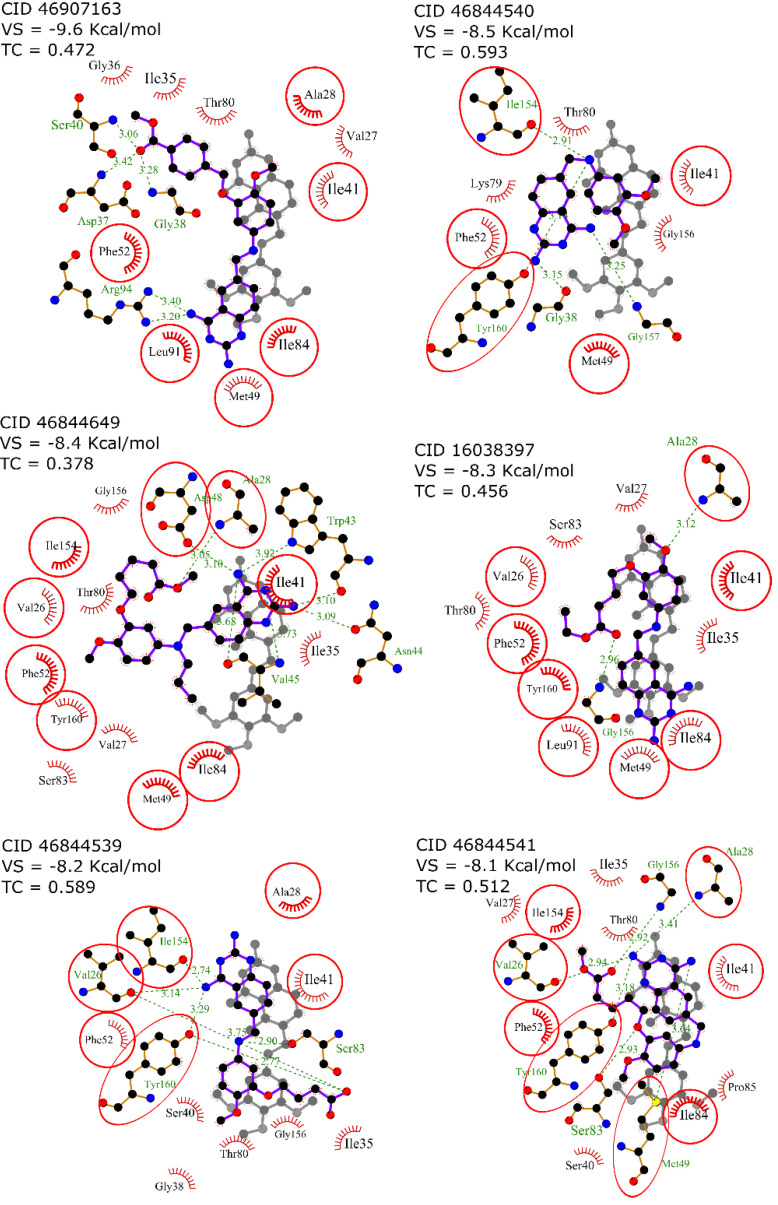
2D diagram of docked conformation with the lowest free energy of binding (vina score) of each know inhibitor in comparison with the crystal conformation of trimetrexate. Residues in red circles interacted with both trimetrexate and the docked inhibitor.

An interaction profile is a set of features that describe a protein-ligand complex in terms of their molecular interactions. To determine the interaction profile involved in the inhibition of *Tc*DHFR-TS, six known *Tc*DHFR-TS inhibitors (CID: 16038397, 46844539, 46844540, 46844541, 46844649 and 46907163) were docked against *Tc*DHFR-TS active site using vina and then the interaction profile of each docked complex was generated using PLIP. To illustrate the benefits of using an interaction profile as the basis for our virtual screening in *Tc*DHFR-TS, Figure 3 presents the docked conformation with the lowest free energy of binding (vina’s best-ranked conformation) of each inhibitor in comparison with the crystal conformation of trimetrexate for comparison. It should be noted that despite the similarities in their structures, these conformations are not oriented in the same direction. Therefore, it is not possible to describe a common inhibition mechanism.

**Figure 4:**
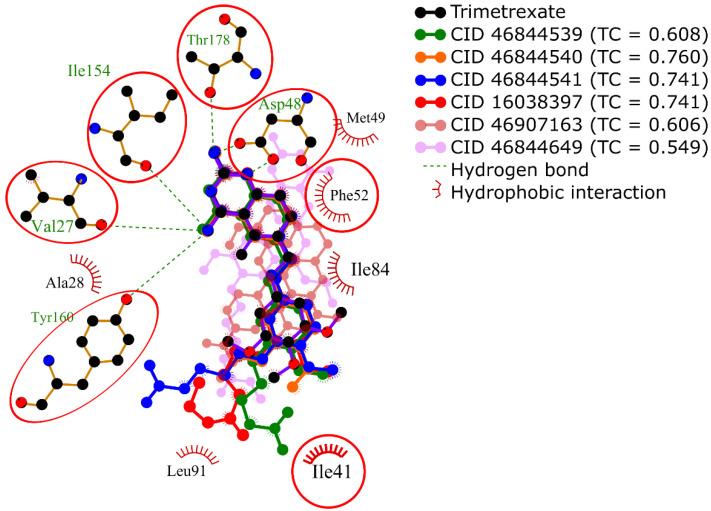
Superposition of 2D diagrams of docked conformations of each known inhibitor with the highest Tanimoto coeffients. Interactions with residues in red circles were highly conserved.

In contrast, Figure 4 shows a different set of conformations whose selection was not based on the free energy of binding but the similarity to the interaction profile of crystal conformation of trimetrexate. This similarity was calculated by determining a set of interaction features of the docked conformations using PLIP and then comparing these features with the Tanimoto coefficient. It should be noted that the interaction profiling was better than the docking score in the identification of similar conformations to trimetrexate.

**Figure 5:**
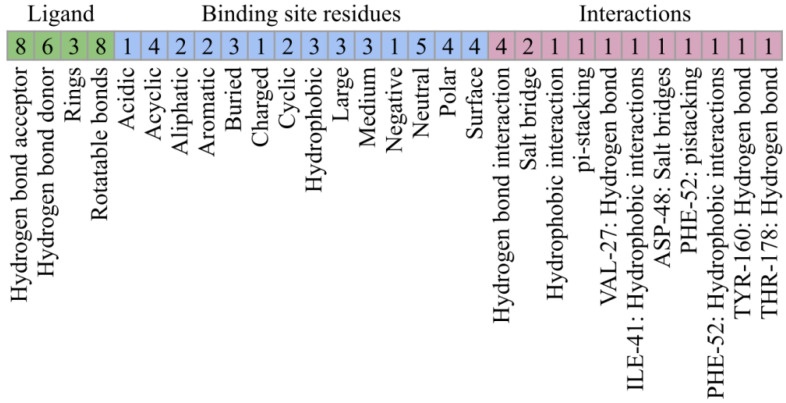
Interaction profile for the inhibition of *Tc*DHFR-TS. Interaction features describe three different aspects of protein-ligand interactions.

For this reason, conformations of the known inhibitors with the highest Tanimoto coefficients were selected to generate the interaction profile present in Figure 5, which describes the common features among the *Tc*DHFR-TS inhibitors. The interaction profile is represented as a set of keys and values, where each key is a unique feature and the value is used for quantitative comparison. This profile was built with the interaction features common in all the known inhibitors, except CID: 46907163 and CID: 46844649 because they did not recreate the trimetrexate orientation properly.

### 2.4. Virtual Screening

**Figure 6:**
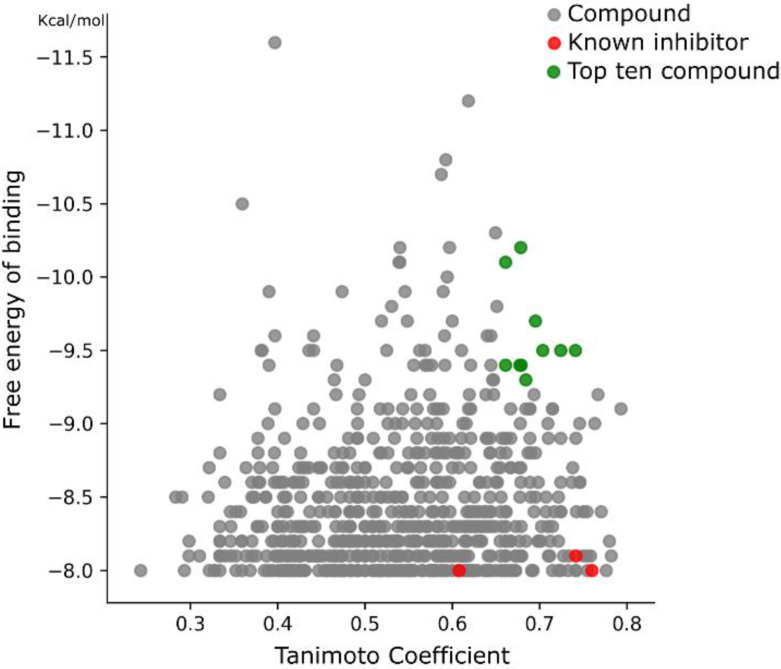
Ranking of compounds from the *Tc*DHFR-TS virtual screening. Compounds in green are the top ten ranked based on both criteria.

A library of 1857 FDA-approved drugs was virtually screened using the free energy of binding estimation and the interaction profile calculation to identify new potential *Tc*DHFR-TS inhibitors. To perform this screening, each ligand was docked in the *Tc*DFHR-TS binding site using vina. Based on the docking results of trimetrexate and the known inhibitors, a cutoff of −8.0 Kcal/mol was set and all the conformation with a vina score below this value was discarded. For each remaining conformation, an interaction profile was generated. The selection of the most promising compound was achieved using the Tanimoto similarity between each FDA-drug profile and the interaction profile calculated from the known inhibitors. The Tanimoto coefficient has a range between 0 and 1, where 1 is the highest similarity and 0 is the lowest. All duplicate ligands were removed keeping only the conformation with the highest similarity. The combined ranking of the Tanimoto coefficient and the vina score led to the plot in Figure 6. This plot shows the vina score ranking from bottom to top, where the dots in the highest part of the plot are the best-ranked compounds by vina. The Tanimoto coefficient is presented from left to right, where the dots on the right are the compounds with the highest similarity with the known inhibitors. The red dots in the plot represent the known *Tc*DHFR-TS inhibitors. Strikingly, the known inhibitors showed poor docking scores, but very good Tanimoto scores, highlighting the importance of the interaction profiling analysis. Among those compounds with a high Tanimoto score there was variation regarding the vina score. Lastly, the compounds in green were considered the top ten potential inhibitors of *Tc*DHFR based on both rankings. This selection considered a high similarity in their interaction features with the known inhibitors and a high affinity from the vina docking.

In Table 3 the top ten compounds of the virtual screening are shown. They showed substantial heterogeneity in their pharmacological applications. Nebivolol, a beta-1 adrenergic receptor antagonist was the best-ranked compound. Three drugs, glipizide, glyburide, and gliquidone are used in the treatment of diabetes as hypoglycemic drugs and have a similar moiety structure. Two kinase inhibitors, nilotinib, and imatinib are used for the treatment of some types of cancer. The drugs dihydro-alpha-ergocryptine and dihydroergocornine, are both used in the treatment of Parkinson’s. Finally, darifenacin, a medication used to treat urinary incontinence, and eltrombopag, which is used in the treatment of chronic immune thrombocytopenia.

### 2.5. In Vitro Activity

**Figure 7:**
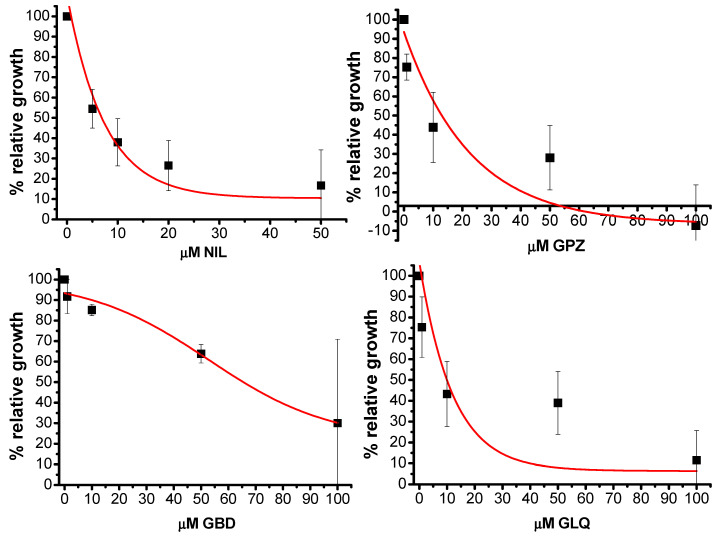
Mean and standard deviation of the effects of nilotinib (NIL), glipizide (GPZ), glyburide (GBD) and gliquidone (GLQ) on the growth of *T. cruzi* epimastigotes after 24 h.

Based on the computational insight the FDA-approved drugs nilotinib (NIL), glipizide (GPZ), glyburide (GBD), and gliquidone (GLQ) were tested in vitro against *T. cruzi* epimastigotes. The effects on the relative growth of the parasites are shown in Figure 7.

NIL was the compound with the lowest IC_50_ (6 ± 2 μM). GPZ and GLQ had a similar inhibition activity with IC_50_= 13.4 ± 6 μM and IC_50_ = 12 ± 5 μM, respectively. Finally, GBD had an IC_50_= 66 ± 12 μM. For comparison, under similar exposure protocols, the IC_50_ for benznidazole was 12 ± 2 μM [35] whereas for NFX the value was 3 ± 0.6 μM (Aketzalli Silva Carmona & Emma Saavedra, unpublished results). In addition, the compounds were evaluated on human foreskin fibroblasts (HFF1) to measure cytotoxicity; the effects on these cells are summarized in Table 4.

## 3. Discussion

In order to identify new potential *Tc*DHFR-TS inhibitors, the structure of this enzyme in complex with NADPH and the inhibitor trimetrexate was analyzed by docking and PLIP. First, trimetrexate was redocked in the *Tc*DHFR-TS active site. Docking was performed treating the residues as rigid. This setup generated that the docked conformation had an RMSD of 2.451 Å compared to the crystal. Nevertheless, interactions were well represented through docking. To generate the interaction profile of known inhibitors against the *Tc*DHFR-TS active site, the interaction features of each inhibitor were calculated and then the mean values of each feature were used. The features were classified into three categories. In the first category, ligand structure features did not show many differences with trimetrexate since all the inhibitor structures were derivatives. Next, interacting residues were evaluated. Most of the residues describing this portion of the interaction profile were neutral, only ASP-48 (negatively charged) was observed. This residue provided an important salt bridge and therefore a major hydrophilic interaction for inhibition [36]. Additionally, there was no substantial difference between the number of hydrophobic and polar residues in the interaction profile. Nevertheless, the *Tc*DHFR-TS active site was described as dominantly hydrophobic [34], mainly due to the presence of two aromatic residues, PHE-52 which could be targeted through π-stacking and PHE-88, whose equivalent residue in human dihidrofolate reductase (*Hs*DHFR) was ASN [37]. Finally, binding present in the interaction profile showed a high number of hydrogen bonds compared to the rest of the interaction types; however, previous work indicated that hydrogen bonding contribution to the binding energy was weak [37].

According to the docking calculation, nebivolol binds to *Tc*DHFR-TS through π-stacking with PHE-52, four hydrogen bonds with ALA-28, GLY-156, GLY-157 and TYR-16, and hydrophobic contacts with ILE-41, PHE-52, LYS-79, and TYR-160. Although it contains two fluorine atoms they seem not to interact in the binding site. This compound contains two chromane moieties in its structure. Chromane derivatives can bind to *Hs*DHFR [38]. They have also been proposed as antiprotozoal agents exhibiting high activity against *T. brucei*, *L. donovani* and *P. falciparum* [39].

On the other hand, nilotinib formed a halogen bond with ILE-35. The rest of the interactions were mainly hydrophobic. It had contact with ILE-41, LYS-79, THR-80, PRO-85, and PHE-88. In the case of imatinib, it only formed one hydrogen bond with 154 ILE. It had a hydrophobic interaction with ILE-35, ILE41, PHE-52, and 2xILE84, and had a π-stacking interaction with PHE-52. Nilotinib and imatinib are tyrosine kinase inhibitors. These compounds have been tested on several nonkinase targets [40,41,42]. Moreover, in a static-cidal assay, nilotinib has shown some activity against the trypomastigotes of *T. cruzi* Silvio X10/7 A1 strain [43]. Additionally, imatinib and some derivatives have been tested against Tulahuen strain [44] showing the potential use of these compounds alone or in combination with benznidazole, the standard treatment for Chagas disease.

Glipizide interacted with ALA-28, SER-83 and GLY-156 by hydrogen bonding. It also had hydrophobic interaction with ILE-41, PHE-52, PHE-88 and LEU-91. It is a sulfonylurea used in the treatment of type 2 diabetes mellitus. Although there is no report of glipizide or derivatives against *T. cruzi* or DHFR, the sulfonyl group is much used in drug design and some DHFR inhibitors contain a sulfonyl moiety [45,46]. Glyburide interacted with ALA-28, SER-83, ILE-154, GLY-156, and TYR-160 by hydrogen bonding and had hydrophobic interaction with ILE-35, ILE-41, and THR-80. It also formed π-stacking with PHE-52. Glyburide has shown anti-leishmanial activity, where binding to *L. donovani* Trypanothione synthetase was suggested [47]. For gliquidone, hydrogen bonding occurred with ALA-28, GLY-156 and TYR 160. It had hydrophobic interaction with ILE-35 and THR-80, and π-stacking with PHE-52. This compound has been proposed to target *T. cruzi* cruzain based on a computational approach, but there is a lack of experimental evaluation [48].

Dihydro-alpha-ergocryptine is a dopamine D2 receptor agonist. It interacted with *Tc*DHFR-TS with ALA-28 and TYR-160 by hydrogen bond and with hydrophobic interactions with ILE-35, ILE-41, THR-80, ILE-84, and PHE-88. Dihydroergocornine, a serotonin receptor antagonist, formed hydrogen bonds with ALA-28 and hydrophobic interactions with ILE-41, PHE-52, THR-80, SER-83, ILE-84, and TYR-160. Darifenacin had hydrophobic interactions with VAL-26, ILE-35, ILE-41, PHE-52, THR-80, ILE-84, and TYR-160 and formed a hydrogen bond with ILE-41. This compound is a benzofuran derivative; interestingly, this kind of compound has been proposed as anti-*T. cruzi*, acting on the mitochondrial electrochemical membrane potential [49].

Finally, eltrombopag is a biphenyl carboxylic acid derivative that targets the thrombopoietin receptor. It formed a hydrogen bond with THR-80 and had hydrophobic interactions with hydrophobic ILE-35, ILE-41, PHE-52, ILE-84, PHE-88, and TYR-160.

Most of the compounds showed interaction with IlE-41, and PHE-52, these residues corresponded to the interaction profile of the known *Tc*DHFR-TS inhibitors. Additionally, ALA-28, THR-80 and TYR-160 are also frequent in the selected compounds. Due to the importance of these residues in the binding of NADPH, these compounds could avoid the function of the co-factor. In vitro testing showed that nilotinib, glipizide, glyburide and gliquidone had activity against *T. cruzi* epimastigotes in the micromolar range. Although they seemed to have an effect on HFF1 cells, these compounds could be used as a starting point for further lead optimization.

## 4. Materials and Methods

### 4.1. Protein Structure Preparation

The crystal structure of *Tc*DHFR-TS in complex with trimetrexate (PDB ID: 3HBB) was obtained from the PDB [50] (www.rcsb.org). Next, the protein structure was extracted, missing side chains were repaired, and all hydrogens were added with the Dock Prep tool of UCSF Chimera [51]. The residues were treated as rigid and the script prepare_receptor4.py from MGTools 1.5.6 [52] was used to add AutoDock atom types and charge to the prepared protein structure.

### 4.2. Ligand Preparation

**Figure 8:**
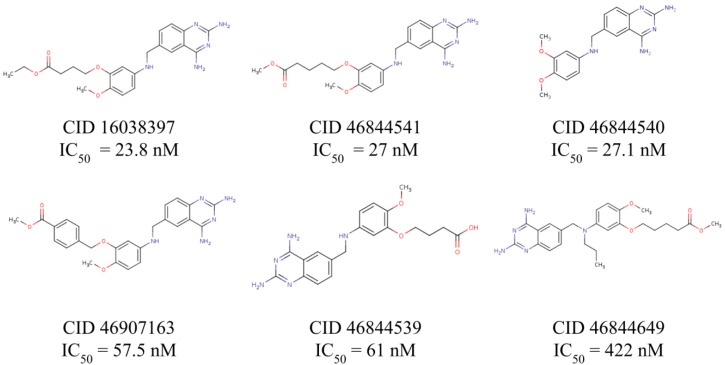
Known *Tc*DHFR-TS inhibitors.

A library of 2355 approved-drugs was obtained from DrugBank [53]. The Open Babel tool [54] was employed to select unique structures from the library and minimize them. The MGTools script *prepare_ligand4.py* was employed to assign charge and atom types from Autodock to each ligand structure. Due to uncommon atom types and a high number of rotatable bonds, only 1857 of these ligands were considered for this work. In addition six known inhibitors of *Tc*DHFR-TS: Ethyl4-(5-[(2,4-diamino-6-quinazolinyl)methyl]amino-2-methoxyphenoxy)butanoate (CID 16038397); Methyl5-(5-[(2,4-diamino-6-quinazolinyl)methyl]amino-2-methoxyphenoxy)pentanoate (CID 46844 541); 6-[(3,4-Dimethoxyanilino)methyl]-2,4-quinazolinediamine (CID 46844540); Methyl4-[(5-[(2,4-diamino-6-quinazolinyl)methyl]amino-2-methoxyphenoxy)methyl]benzene carboxylate (CID 46907163); 4-(5-[(2,4-diamino-6-quinazolinyl)methyl]amino-2-methoxyphenoxy)butanoic acid (CID 46844539); methyl5-{5-[[(2,4-diamino-6-quinazolinyl)methyl](propyl)amino]-2-methoxyphenoxy}pentanoate (CID 46844649) were retrieved from PubChem. These compounds are summarized in Figure 8.

### 4.3. Molecular Docking

Molecular docking was performed using AutoDock Vina 1.1.2. According to the crystal structure of the trimetrexate-*Tc*DHFR-TS complex (PDB ID: 3HBB), docking was performed in the coordinates of the binding site: X = 21.8, Y = 39.5, Z = 25.0.

### 4.4. Interaction Profiling

An interaction profile is a set of keys and values that represents the interaction features occurring in a complex protein-ligand. The keys represent each feature and the value is a quantitative measure of the feature. The interaction features are based on the structure features of the ligand, the residue properties of the interacting residues and the noncovalent intermolecular interactions with the *Tc*DHFR-TS binding site. These interactions were calculated with the Protein-Ligand Interaction Profiler (PLIP) python package [33].

### 4.5. Similarity Calculation

The interaction profiles were compared using the Tanimoto coefficient for similarity calculation [55]. This coefficient was calculated using the equation:

Tanimoto coefficient = A∩B/(A + B − A∩B)(1)

where A∩B is the sum of the features common in both profiles, A is the sum of the features present in profile A, and B is the sum of the features present in profile B.

### 4.6. Cell Culture

HFF1 fibroblasts (human foreskin fibroblasts) were seeded in Petri dishes (60 mm) with fresh DMEM medium (Dulbecco’s Modified Eagle Medium, Gibco, USA: 25 mM d-glucose, 4 mM l-glutamine, 0.03 mM phenol red, 5.3 mM KCl, 110.3 mM NaCl) supplemented with 10% fetal bovine serum (FBS; Biowest, South America) and antibiotic (100 µg streptomycin/mL and 100 U penicillin/mL, Sigma-Aldrich, USA), and incubated at 37 °C and 5% CO_2_ up to 100% confluence.

Epimastigotes of *T*. *cruzi* (TBAR/MX/0000/Queretaro strain) were grown in LIT culture medium (Liver Infusion-Tryptose: 0.5% liver infusion, 0.5% tryptose, 0.42% sodium phosphate, 0.4% NaCl, 0.2% glucose, 0.04% KCl) supplemented with 10% of FBS, hemin (0.025 mg/mL) and antibiotic (100 µg streptomycin/mL and 100 U penicillin/mL, Sigma-Aldrich, USA), were incubated at 28 °C.

### 4.7. Exposure to FDA-Approved Drugs

HFF1 cells in the exponential phase of growth were harvested and 1 × 10^4^ cells were seeded per well in microplates in 0.2 mL DMEM medium. They were incubated 24 h at 37 °C for adherence. They were subsequently exposed to different concentrations of the FDA-approved drugs (0−100 µM added in a maximum volume of 2 µL resuspended in DMSO). Control cells without drugs were added with 2 µL DMSO. They were incubated for 24 h. After this time, viability was determined with 0.4% trypan blue and counting in a Neubauer chamber.

Epimastigotes of *T. cruzi* in the exponential phase of growth were counted with a Neubauer chamber, 2 × 10^5^ parasites were seeded per well in a volume of 0.2 mL of LIT medium. They were subsequently exposed under the same conditions as HFF1. They were incubated for 24 h at 28 °C and viability (mobile parasites) was determined in a Neubauer chamber.

The IC_50_ was determined by plotting the % growth at 24 h of exposure against the concentration of the compound. The experimental data were fitted to the dose-response function of the Origin 8 software according to the equation:

y = A_1_ + (A_2_ − A_1_)/(1 + 10^(log X0 − X)p^) (2)

where X0 corresponds to the IC_50_ value, X values are log of concentration, and p here is the Hill coefficient or fitting, the A2 value was fixed to 100% relative growth (at 0 mM of the compound) and the p value was variable.

### 4.8. FDA-Approved Drugs

Nilotinib (NIL, CDS023093), glipizide (GPZ, G117), glyburide (GBD, PHR1287), and gliquidone (GLQ, CDS021537) were acquired from Sigma-Aldrich, Toluca, México.

## 5. Conclusions

In the current study, a virtual screening methodology based on docking, and an interaction profile of trimetrexate and six other known *Tc*DHFR-TS inhibitors for the repositioning of FDA-approved drugs was designed. This study provided an insight into the interaction features responsible for the inhibition of *Tc*DHFR-TS, which led us to propose ten potential new inhibitors for this enzyme. Among them, the multitarget kinase inhibitors, imatinib, and nilotinib have previously reported activity against *T. cruzi*. Glipizide, glyburide, and gliquidone, which are anti-diabetic compounds, also seem to have the structural features needed to bind to *Tc*DHFR-TS. In vitro studies indicate that nilotinib and these three compounds had an inhibitory effect on the growth of *T. cruzi* epimastigotes. Therefore, these results could lead to more efficient anti-Chagas treatment, through lead optimization and analog screening.

## Figures and Tables

**Table 1:** Main interactions of the trimetrexate-*Tc*DHFR-TS complex.

Ligand Properties		Interacting Residues Properties		Interaction Pattern	
Hydrogen bond acceptor	8	Acidic	1	Hydrogen bonds	4
Hydrogen bond donor	5	Acyclic	4	Hydrophobic interactions	2
Rings	4	Aliphatic	2	Salt bridges	1
Rotatable bonds	6	Aromatic	1	hb ^1^: TYR-160	1
		Buried	2	hb: THR-178	2
		Charged	1	hb: VAL-26	1
		Cyclic	1	hi ^1^: ILE-41	2
		Hydrophobic	2	sb ^1^: ASP-48	1
		Large	2		
		Medium	3		
		Negative	1		
		Neutral	4		
		Polar	3		
		Surface	4		

^1^ hb = hydrogen bond; hi = hydrophobic interaction; sb = salt bridge.

**Table 2:** Values of RMSD and AutoDock Vina (vina) score of trimetrexate conformations on the active site of *Tc*DHFR-TS.

Conformation	RMSDÅ	Vina Score Kcal/mol	hb ^1^: TYR-160	hb: THR-178	hb: VAL-26	hi ^1^: ILE-41	sb ^1^: ASP-48
Crystal	-	-	1	2	1	2	1
1	3.044	−8.5	1	1	-	-	1
2	10.810	−8.4	2	1	-	1	1
3	3.768	−8.4	1	1	1	-	1
4	10.762	−8.4	2	1	-	1	1
5	3.605	−8.1	1	-	-	-	1
6	2.451	−8.0	1	1	1	1	1
7	8.232	−7.9	-	-	-	-	-
8	7.916	−7.9	-	-	-	-	-
9	7.592	−7.8	-	-	-	-	-

^1^ hb = hydrogen bond; hi = hydrophobic interaction; sb = salt bridge.

**Table 3:** Top ten food and drug administration (FDA)-approved drugs in the *Tc*DHFR-TS virtual screening based on their interaction features and free energy of binding.

Name	Structure	Vina Score Kcal/mol	Description
Trimetrexate	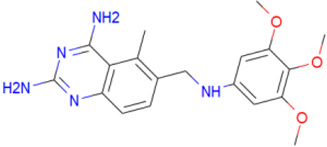	−8.5	DHFR-TS inhibitor
Nebivolol	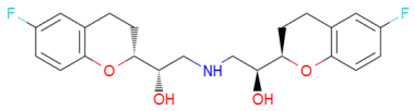	−10.2	Treatment of hypertension
Nilotinib	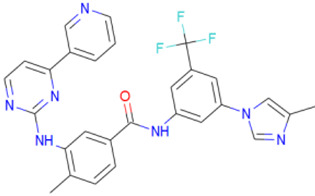	−10.1	Tyrosine kinase inhibitor
Glipizide	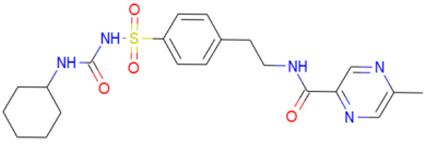	−9.8	Anti-diabetes drug
Glyburide	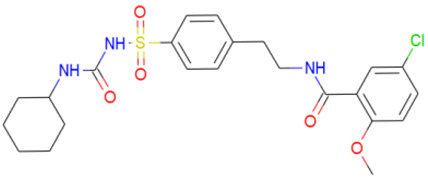	−9.7	Anti-diabetes drug
Gliquidone	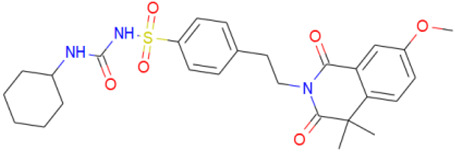	−9.5	Anti-diabetes drug
Imatinib	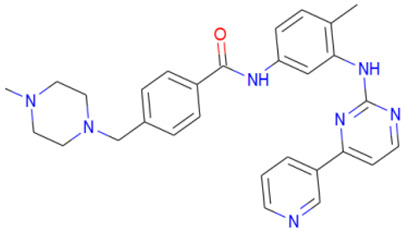	−9.5	Tyrosine kinase inhibitor
Dihydro-alpha-ergocryptine	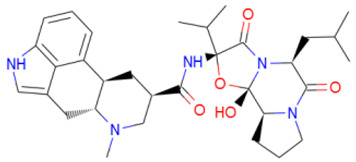	−9.5	Early treatment of Parkinson’s disease
Dihydroergocornine	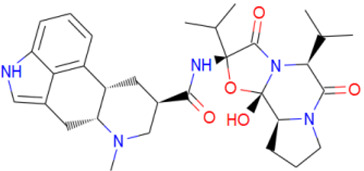	−9.4	Early treatment of Parkinson’s disease
Darifenacin	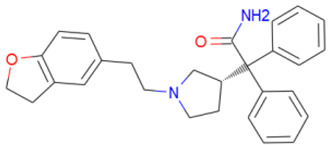	−9.4	Treatment of urinary incontinence
Eltrombopag	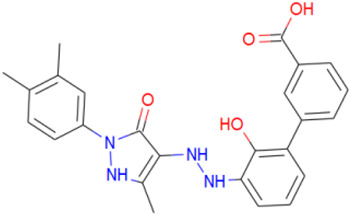	−9.3	Treatment of chronic immune thrombocytopenia

**Table 4:** Half maximal inhibitory concentration of potential *Tc*DHFR-TS inhibitors against *T. cruzi* and HFF1.

Compound	IC_50_ μM *T. cruzi*	IC_50_ μM HFF1
NIL	6 ± 2	12 ± 6
GPZ	13.4 ± 6	38 ± 11
GLQ	12 ± 5	68 ± 14
GBD	66 ± 12	>50

## Abbreviations

DHFR	Dihydrofolate reductase
TS	Thymidylate synthase
NIL	Nilotinib
GPZ	Glipizide
GLQ	Gliquidone
GBD	Glyburide
